# Exploring Behavioral Readiness and Program Strategies to Engage Older Community Residents in Advance Care Planning: A Pilot Mixed-Method Study in Taiwan

**DOI:** 10.3390/ijerph17124285

**Published:** 2020-06-16

**Authors:** Hsin-Lung Chan, In-Fun Li, Ling-Chun Tseng, Yvonne Hsiung

**Affiliations:** 1 Division of Family Medicine, Mackay Memorial Hospital, Taipei 104, Taiwan; hunter5770@gmail.com; 2 Department of Nursing, Tamshui Branch, Mackay Memorial Hospital, New Taipei City 251, Taiwan; lif1129@mmh.org.tw; 3 Department of Nursing, Tai-Tung Branch, Mackay Memorial Hospital, Tai-Tung 950, Taiwan; a1449@mmh.org.tw; 4 Department of Nursing, Mackay Medical College, New Taipei City 252, Taiwan

**Keywords:** readiness for advance care planning, older community residents, Taiwanese, palliative care, intervention strategies

## Abstract

Taiwan was the first Asian country to endorse patient autonomy, and advance care planning (ACP) has been highly promoted to improve quality of end-of-life (EOL). A mixed-methods pilot survey was conducted in northwestern Taiwan to investigate older community-dwelling residents’ (N = 52) ACP behavioral engagement, socio-demographical correlates, and their preferred intervention strategies. An interview subset (25%, N = 13) was purposely chosen for in-depth feedback and rationales behind their ACP decision-making. Rich information was obtained about perceived facilitators and inhibitors to initiate ACP and preferred intervention strategies in ACP programs. Consistent with previous literature, carefully designed ACP programs that incorporated family decision-making and met older subjects’ multiple needs would increase program acceptability and foster ACP engagement among older Taiwanese in the community setting.

## 1. Introduction

Advance care planning (ACP) is a process that aims to support patients’ medical decisions in the event of incompetency [1,2]. ACP has been associated with positive patient and caregiver outcomes, including increased quality of remaining life, improved bereavement process, and enhanced psychological well beings [3]. Advanced discussions and practical preparations for “in-the-moment” end-of-life (EOL) decisions (p. 256) [4] provide guidance to family and surrogates, decrease their decisional burdens, and reduce health care providers’ moral distress [5]. As illness evolves, patients will be assured by documented ACP discussions to receive EOL care consistent with their goals, values, and preferences [4]. However, contextual factors influencing ACP engagement are often complex, “reflecting the diverse and often competing needs of patients, health professionals, legislature, and health systems” (p. 1026) [6].

Individuals’ ACP updates vary across settings and populations, between 7.8% and 60%, particularly higher among western mainstream cultures [7]. However, even among Caucasian American patients with advanced cancer [8], ACP is not executed as often as advance directives (ADs). Between 2011 to 2016, approximately 38.2% American patients with chronic illnesses and 32.7% healthy adults have completed their ADs, but only some have ACP awareness [9]. Despite a patient-centered e-ACP (PREPARE) program that successfully increased ADs completion from 25% to 35% [10], documented ACP discussions were below 20%. In multicultural countries, individuals of minority ethnic backgrounds generally have lower ACP uptakes than the mainstream groups [11,12]. When explaining this wide range of acceptability in a global medical context, the universal applicability of ACP is often debated, where the two cultural issues in non-white cultures are considered, namely, collective (family-centered) versus autonomous (patient-centered) approaches, and patient preference for who should be involved in EOL decision-making [12].

ACP uptake in Taiwan is between 10–12% [13,14]. Taiwan was the first country in Asia to promote patient autonomy of the terminally ill for their quality of dying. In 2000, Taiwanese patients were given legal rights under the amended “Hospice Palliative Care Act [15],” for EOL decisions. While the seeds of palliative care have taken root, the Patient Right to Autonomy Act in 2019 further entitled all Taiwanese of unbearable and suffering conditions for their own life-support, including patients of terminal illness, irreversible coma, permanent vegetative state, severe dementia, and other disease conditions [16]. This noticeable social movement in Taiwan highlighted the value of life sanctity and endorsed every Taiwanese citizen’s ACP [17]. In Mandarin Chinese, ACP stands for “autonomous medical plan”, a phrase to intentionally avoid deaths with a positive connotation. In order to officially appoint medical attorneys or transliterate AD for future incompetency, ACP is required to be documented in the national health database. In Taiwan, fees of ACP are not covered by the national health insurance, and such sessions can only be conducted by trained physicians, nurses, clinical psychologists, and social workers [17].

Although efforts have been made by the government to support palliative care, the general stage of the Taiwanese’s readiness for ACP is still in its infancy. Results from a nationwide Taiwanese survey in 2010 showed that ACP was not commonly mentioned among oncology care physicians and nurses, and their own ACP engagement was largely influenced by knowledge and attitudes related to the Hospice Palliative Care Act [18]. Whereas dignified deaths have become the eminent doctrine in the Taiwanese medical context, older Taiwanese rarely initiate ACP [13], in spite of their inevitably needs to face EOL and decreased functioning abilities. This group did not foresee deaths in the near future as a likely outcome, felt no right to make treatment decisions for themselves, and lacked motivation to plan for EOL [13,19,20]. Several Taiwanese studies [19,20,21] have established the associations between AD completion and life-support utilization among older Taiwanese [22,23], but up to now little is known about ACP behavioral engagement.

To ultimately increase ACP uptakes, this pilot study made an initiative to assess readiness for ACP, discover significant socio-demographical correlates, and investigate program strategies preferred by older community-dwelling residents. A mixed-method design was employed in which both qualitative and quantitative findings collaborated to explore facilitators and inhibitors that possibly influence subjects’ ACP engagement.

## 2. Materials and Methods

### 2.1. Study Design and Subjects

Approved by the Mackay Memorial Hospital Institution of Review Board (Approval of Human Ethics #15MMHIS12e7), this descriptive study was part of the “Northwestern Taiwanese Residents’ Health Examination Survey” and an exploratory triangulation design (QUAN to qual) [24] was employed. The rationales of using a mixed-method approach [25,26] were to illustrate “complementarity” and “development” [27] in the pilot stage. In our case, the phenomenon to be explored was the emphasis on the lived experiences of older Taiwanese community residents’ ACP decision-making and their behavioral engagement.

Our data were collected from two government-contracted health examination centers in northern Taiwan. Potential subjects (≥65-year-old) were already in the national geriatric registry and recruitment flyers were posted with participation criteria at the time of free health check-ups. The inclusion criteria for this pilot study were self-identified Taiwanese in the community. Specifically, they were aged and above 65-year-old, currently un-institutionalized, legally competent for making life-support decisions, and communicable in Mandarin and/or Taiwanese about their current medical conditions and EOL preferences. Excluded were those who were critically ill, hospitalized, institutionalized (in nursing homes or hospices), legally incompetent for ACP decisions, and/or unable and reluctant to discuss ACP matters during the data collection period. Specific attention was paid for the influence of gender in this cross-sectional study, yet no evident patterns were found in the final sample. There was no noticeable gender difference in the interview subset as well.

### 2.2. Study Ethics and Procedures

Older community-dwelling Taiwanese subjects eligible for this study were immediately approached, and those agreed to discuss their ACP engagement were invited to a separate waiting room. After they completed their annual health examinations, written information was provided about this pilot survey. The anonymous and voluntary (no participation fees) nature was verbally explicated before obtaining oral informed consents. All survey data were de-identified and separated from their health examination in the hospital database. While subjects’ risks for participating in this study were no greater than those who did not participate, the ethical approval was granted for an institutional data analysis in which subjects’ signatures were waived.

Within the 6 months of data collection, sixty-six potential patients were approached. Among them, six (9%) were excluded due to their nursing home residency. Eight qualified individuals (12.1%) declined to participate, allegedly due to unavailability. None of these potential older community residents reported being offended by the survey topic. Each older Taiwanese in the final sample (N = 52) completed a bespoke type of questionnaire [28] established by the institution, and a total of 13 subjects were purposely selected (25% of the sample) to be individually interviewed for 15 to 30 min on the same day. Interview subjects were encouraged to freely express their interests and concerns about ACP and possible participation in intervention programs.

### 2.3. Quantitative Measures and the Interview Guide

Based on behavioral change constructs of Prochaska’s Transtheoretical Model (TTM) [29,30], inquiries were developed to assess subjects’ stage of readiness and preferred strategies for ACP. Survey questions were derived from previous research among older Chinese Americans [22,23,31] but modified for this pilot study under the Taiwanese legal and medical context. The questionnaire was comprised of a series of demographical questions, a behavioral readiness measure, and multiple-choice questions about program strategies, including their preferred group size, company, activities, length, frequency, and intervention location. Specific attention was given in the interview to seek for factors older subjects believed to facilitate and inhibit their ACP engagement.

In order to precisely identify each subjects’ readiness for ACP, a 7-item algorithm, rather than a summative scoring, was originally created according to the TTM stipulations to assess ACP behavioral readiness among Chinese-ethnic immigrants [22] (Table 1). This measure was inspired by an observational cohort study in which older persons’ engagement in ACP and factors associated with readiness to participate in ACP were characterized and conceptualized by a series of stage-to-change questions.

To account for older subjects’ education levels and survey burdens, questions were written in traditional Chinese at the fifth-grade literacy level, and survey readability was originally tested by two elementary school students. Older Taiwanese’s ACP engagement was on a theoretical basis, for example, if one has an AD legally completed more than six months ago and still continued to communicate his/her EOL preferences with family members, he/she would be placed in the most advanced stage of change, maintenance. On the contrary, if subjects had a lack of awareness (i.e., they never heard of any ACP concepts), an avoidance (i.e., they had no interest in ACP), and/or a disbelief of such planning, they would be placed in the pre- contemplation stage. The final score of the stage of change measure ranged from “precontemplation (non-believers = 1 and believers = 2)” to “maintenance (6)”. The higher the stage score, the better the behavioral engagement. In this study, two nurse professors with palliative care expertise reviewed the algorithm scores and each subject was examined and manually positioned in one stage of readiness.

More than 1400 Taiwanese patients from different clinical settings have used this algorithm; older Chinese-ethnic Americans and Taiwanese in Asia both reported good clarity and ease of use. In this study, older Taiwanese generally completed the survey within 15 min without any confusion and the algorithm tool served well in distinguishing each subject’s readiness in one of the six stages for their ACP engagement. Good internal consistency was reported (Cronbach’s alpha = 0.92, *p* < 0.01), slightly higher than it was in the original older overseas population (Cronbach’s alpha = 0.86, *p* < 0.01) [10].

Our semi-structured interviews, a traditional form to collect qualitative data [32,33], focused on clarifying survey answers and further explored subjects’ rationales behind ACP decision-making, modified from a previous ACP study among older Chinese American immigrants [22]. Interview subjects were encouraged to provide socio-cultural, knowledge, spiritual, and demographic factors that they believed to influence their ACP engagement. However, our interview guide was revised to concentrate on the facilitators and inhibitors after reviewing preliminary quantitative findings from the survey and during the first four interviews.

All interview questions were open-ended which allowed the subjects to freely share personal stories and acquaintance with the EOL experience of others. Simple explanations of ACP concepts, such as life-sustaining treatment and ADs were introduced in a standard format in the guide to avoid medical jargons and possible distortion. For example, instead of using the term ACP, the interviewer explained this term by stating, “In Taiwan, people are encouraged to make life-support decisions about treatment and care when becoming seriously ill. There are clinics that you may pay for such guided discussions. We would like to know your plan should you become too ill to let others know of your preference at the time.” The final version of the interview guide consisted of the following sections: (1) an introduction section that allowed the researcher to get acquainted with the subject; (2) an assessment of ACP readiness, which included an understanding of their motivation status, pros and cons, and barriers specifically perceived related to ACP; (3) recommendations for ACP intervention strategies; and (4) reflections on the interview.

### 2.4. Quantitative and Qualitative Data Analyses

All survey data were entered manually into SPSS Statistics for Macs, Version 19.0 (IBM Corp, Armonk, NY, USA) for descriptive analyses. The final interview subset contained 6 males and 7 females whose answers were further clarified and coded, searching for the rationales behind ACP decision-making. Since the purpose of obtaining qualitative data from the older Taiwanese community sample subset was to support, clarify, augment, and possibly explain the quantitative survey data, in order to maximize variations, our interview subset (n = 13) was selected based on their demographical profiles and readiness (Table 2).

Qualitative data management analysis [34,35] involved four phases: processing and translating data, constructing a code list and applying codes to the data, describing cases and identifying themes, and ensuring quality of data. Interview data analysis was initiated soon after the two pilot interviews were obtained. For further content analyses [34], all verbatim responses from interview subjects were organized and entered into ATLAS.ti 5.0 Software (Scientific Software Development GmbH, Berlin, Germany). A deductive qualitative approach was adopted by two independent researchers/reviewers who have palliative care expertise. The final result was approved by a senior oncology nurse professor who reviewed and agreed the overarching main categories across cases.

## 3. Results

### 3.1. Older Taiwanese Community Residents’ ACP Engagement

#### 3.1.1. Sample Characteristics

All fifty-two subjects resided within a 20-mile radius of the study site in northwestern Taiwan. One-fourth (N = 13) of them were purposely selected and individually interviewed for 25–40 min by one nurse researcher. The interview subset has a maximized socio-demographic variability to represent the whole sample (Table 2).

#### 3.1.2. The Ordinal and Dichotomous Stages of Readiness for ACP

Each older subject was assigned to one of the six behavioral stages of readiness, according to their intentions for ACP, completion of advance directives, and status of EOL communication (Table 1). Only two (3.84%) had no interest in further learning and disbelieved ACP benefits. The majority heard about ACP for the first time but generally the idea that, “Making my EOL wishes known to others in advance is great” and also, “Oh no, bad things will not happen after initiating ACP; this is just a traditional myth.” In spite of a good ACP intention, ADs completion in this sample was low. Only one did so but she had no motivation to further share EOL preferences with her physician or significant other. Most older subjects admitted having no definite timeline to proactively contemplate, prepare for, or initiate ACP discussions in a foreseeable future; without further facilitations, only a few (15.38%) can be expected for ACP behavioral changes within six months (Table 3).

Our pre-contemplators also demonstrated lower self-efficacy than others in the communication aspect; they admitted being moderately to highly influenced by significant others whether to initiate ACP. While the Taiwanese cultural custom is a shared decision-making model within the family, the concept of family durable power of attorney for health (DPOA) is nonetheless strongly endorsed. “I definitely want my husband(wife) to decide for me.” However, many older subjects in fact reported their significant family members, unclear about their wishes, probably had no interest in serving as decision surrogates at this time point. Discouraging behaviors were also described during interviews, such as their EOL conversations being interrupted or stopped by their family members, “They think this topic is too negative… no need to mention this now.” Two were interfered by adult children with their wish to complete ADs. Subjects agreed that such discordant opinions in the family might have adversely affected their ACP engagement.

#### 3.1.3. Correlations between Demographical Variables and Stages of Readiness for ACP

To understand correlational relationships between subjects’ demographical characteristics and readiness for ACP (Table 4), appropriate statistics were chosen. Only age and the number of parents deceased (which is also highly age-associated) were found to weakly to moderately (r = 0.36–0.61, *p* < 0.01) associate with ACP engagement. Household income and ACP engagement was weakly correlated (r = 0.23, *p* < 0.05), but this level of income was not significant in the six ordinal stages of readiness.

### 3.2. Participation in ACP Interventions and Preferred Program Characteristics

#### 3.2.1. Interests in ACP Programs

Older Taiwanese subjects’ interest in the ACP program was quantitatively and qualitatively explored, along with preferred strategies that may facilitate their participation (Table 5). The idea of ACP programs was highly rated, and none of the subjects reported being offended by this topic related to death. Participating in a program about ACP know-how would ultimately avoid sufferings at the EOL. Most felt necessary to obtain information for future EOL decisions, but they were unclear about the most appropriate time. About 17% of the sample not interested in a program regarded ACP not a life priority, “simply too busy at this moment…. ACP sounds good but not now. I got no time to go to a program.” Those who had interests were enthusiastic to share the newly learned ACP information and further educated families and friends, “Once I learn what ACP is, I definitely will share with my wife/husband and friends. This is important for people of our age.” However, they too recognized it was difficult to convince the whole family to participate in an ACP program with them.

#### 3.2.2. Strategies Preferred in ACP Programs

Older subjects expected ACP sessions not longer than two hours (86.4%), and in order to gain fundamental ACP knowledge, 40% believed, “A one-time ACP 101 (introductory class) would be just sufficient.” Internet or computer learning was not popular in this age group due to technology limitations and computer unavailability. Among those highly educated and capable of accessing ACP information online, the self-learning mode was favored, “so I am not going to waste my valuable time sitting in a class.” One-on-one ACP sessions were popular since they tailor to individual needs, “I want to be taught by experienced social workers or nurse counselors.” ACP education is not to be offered by physicians because, “I am afraid that’s going to be too intimidating.” A small group of five persons (or fewer) was a recommended size (Table 5), particularly appropriate for discussing specific pros and cons of LST. Some older females admitted their voice would be better heard this way. Breaking the big group into small groups was also suggested as an effective learning strategy, “After ACP 101, we could freely decide whether to continue in the small-group discussions.”

In the multiple-choice section (Table 5), though the majority of older subjects (84.6%) were not concerned with whom took part in the ACP programs, nearly 70% preferred learning with family members, particularly with spouses. A tendency was found in both qualitative and quantitative findings for female subjects to designate husband-surrogates. This is pattern was however not obvious among male subjects. Older Taiwanese also would not want their adult children to be involved in ACP at this point, feeling that their younger generation wouldn’t be interested. “My families are far from ready (for my ACP).” “My children are open to discuss death and dying, they ARE, but it is just unnecessary now and I do not want to bother them.” Those who specifically wanted to choose age-compatible partners desired to share with people of similar values: “Why debate with the youngsters who don’t even understand we old people? They are not old yet, so they don’t know what we think.”.

### 3.3. Influencing Program and Personal Factors of Program Participation

The major facilitator for older Taiwanese to engage in ACP was related to program characteristics. In fact, more than half of the written responses about program facilitators were subjects’ personal expectations and descriptions of an ideal ACP program. Reportedly, a carefully designed intervention would increase ACP readiness, such as resourceful lectures, a complete course agenda, an introduction about the speakers’ background and expertise, learning objectives, suggested readings, and events related to ACP. Rich information was also obtained about the program: older Taiwanese’s ACP engagement would be facilitated if a program was offered free of charge (or inexpensive), with a friendly atmosphere, in a comfortable learning environment, with a feeling of mutual trust between the lecturers and subjects, with a respect for privacy, and in a relaxing pace that they would not need to worry no homework or be forced to speak in the public. Program lecturers and developers were experienced physicians or professors good at teaching and currently practicing in the clinical field.

A self-perceived availability for ACP was reported as an important facilitator, commonly described as, “when I have extra time”, “when I am not busy” or “when I become available.” In other words, subjects would better engage in ACP if “their time allows.” Another category of facilitators was older subjects’ physical condition, self-perceived appropriate to initiate ACP, “When I become older” or “I will have my ACP when becoming sicker.” Older subjects agreed that while they perceived no imminent needs, complicated with the medical uncertainty, it was natural “not to think about EOL now”. Last, older subjects’ willingness to participate in the ACP program rested largely on the availability and convenience of the program; in their words, “effortless.” Subjects admitted that they would “definitely commit better” if the program site was close to their residence or nearby MRT stations.

Regarding the program inhibitors, the majority responded, “no comments”, “no idea”, or “no advice” due to a lack of ACP understanding. However, a pointless or abysmal program was reported to hinder subjects’ willingness to participate in ACP. Descriptions of such an “unworthy-to-go” program were mostly contrasting characteristics mentioned in the above facilitator section. Educational materials being excessively theoretical or too academic would also prevent participation. A continuing participation was determined by the success of the first meeting, “I have to say, the first impression about the program is important”.

A category of reported hindrances of ACP engagement was again about subjects’ self-perceived unavailability (10.2%) and poor physical condition (6%). Interestingly, older subjects used the same reasons to rationalize their ACP decision-making. Six subjects (46%, all >75-year-old) explained in their interview that being healthy was in fact a condition not suitable for ACP, “If I am being physically healthy, why would I go to ACP programs now?” A poor health status was also viewed as a situational inhibitor to prevent ACP, “If I cannot move around, I will not have energy to go to any health programs.” Older subjects resided in the suburban community, and most did not drive and came to the study site alone by public transportations without family members’ company. Although “poor access to the program site” was an inhibitor, transportation was not a general concern according to this relatively healthy sample. The Taipei metropolitan rapid transit was affordable and convenient for older Taiwanese to use in the community.

To summarize, despite the general low readiness for ACP, older Taiwanese in this sample mostly well accepted and believed in the benefits of EOL planning in advance. The majority were interested in an ACP educational program, and specific and certain program strategies were preferred with rationales, such as the need for a short and useful ACP 101 lecture, the format of a small-group sharing and discussion, a self-learning method online, the preferred characteristics of the program contents, etc. The one-fourth interview subset provided details about an ideal ACP program with which they were unfamiliar, yet they anticipated, experienced developers/educators, practical program contents and effective teaching, and support for convenience and accessibility. Other than undesired program characteristics, inhibitors at the personal level included decreased physical functioning and stamina, a lack of awareness or inadequate knowledge, no sense of urgency, future EOL uncertainties, possible troubles and inaccessibility to the program site, and the difficulties of initiating EOL conversations, in particular with spouses and adult children in the family.

## 4. Discussion

### 4.1. Readiness for ACP among Older Taiwanese

Both qualitative and quantitative findings support that the majority of older community-dwelling residents are precontemplator-believers, potential for further ACP behavioral changes [36]. Recognizing that some may be uncomfortable discussing death-related topics, older subjects believe ACP is a good idea, “Early preparation is always good! It is the old Chinese proverb.” Positive attitudes toward ACP also include ACP helps to ensure patient autonomy, protect human dignity, increase a sense of self-control, alleviate possible sufferings at the EOL, facilitate a good death according to one’s desires, help communicate EOL wishes with loved ones, and minimize family decision-making, caring, and economic burdens [10,37]. Their limited ACP awareness and behavioral readiness are comparable to ACP engagement among overseas Chinese-ethnic Americans [22,38] and Australians [39]. Multiple barriers to implementing ACP [14,22,23,31] may explain why most older Taiwanese have no definite timetable to contemplate EOL decisions, no motivations to complete AD documents, or no willingness to initiate any ACP discussions within six months.

#### 4.1.1. Socio-Demographic Influencing Factors to ACP Engagement

Existing international literature shows multiple influencing factors to ACP uptake [7], including socio-demographic factors, ACP comprehension and awareness, cultural aspects, timing of ACP discussions, specific medical conditions, procrastinations, shifts in preferences over time, and the attitudes and beliefs of healthcare providers. Younger age and a lower level of education and income are associated with low ACP uptakes [7]; on the contrary, patients with higher illness and perceived needs are more likely to engage in ACP and consider appointing a surrogate [40]. In our study, increased age and income are positively correlated with ACP readiness—the older and wealthier the Taiwanese are, the more likely they will contemplate ACP. Similarly, those who have lost both parents also seem to be more ready to complete ADs. Historically, ACP is more common among people of higher socioeconomic status and income has direct impact on ACP readiness [7,20]. There is great economic diversity among older Taiwanese and the income status is anticipated to rise along with the level of education. We do not find education a correlate of ACP readiness, whereas subjects’ household income is weakly correlating to ACP contemplation; the effect of income alone on older Taiwanese’s readiness for ACP needs further exploration.

In addition to increased age, female gender is a common correlate of ACP discussions; particularly among urban, community-dwelling overseas Chinese immigrants [22,38], being female is predictive [22,31] in the binary regression model to increase the odds of ACP discussions, but in the correlation results there is usually no specific gender difference in subjects’ readiness outcomes. Similarly, being female is not correlated with ACP engagement, but in our interview, older Taiwanese women emphasize more the importance of future EOL care and how their EOL preferences can be communicated during the ACP process with their loved ones, whereas men’s focus primarily centered on the decisions of AD completions and LST outcomes. Older women seem to be more open and positive in their intention to learn about ACP and psychologically prepared for death. Future research can focus on the attitudinal difference between older males and females in ACP engagement. Although we do not find comparable associations between personal contextual factors of marital status and ACP uptake [22,38], no spouse subjects who live alone may perceive emotional or practical barriers to ACP. Last, we did not investigate subjects’ interaction with healthcare providers, family and friends, while perceived good relationships are suggested as a facilitator to ACP engagement [41], this influencing factor requires future research in Taiwan.

#### 4.1.2. ACP Awareness and ACP Related Knowledge

Low awareness of ACP (never heard about ACP) is reported as one of the major ACP inhibitors [7]. Various ACP knowledge deficits among older peoples are characterized as limited functional literacy (understanding of EOL language), lacked interactive literacy (opportunities for meaningful EOL discussion with providers and families), and insufficient critical literacy (uncertainty about future care) [7,42].

Older Taiwanese subjects’ little ACP comprehension and limited awareness are consistent with previous findings about ACP literacy among older community-dwelling Chinese-ethnic groups. Up to 2016, no ACP discussions were documented in China [43,44], and a 2018 intervention study concluded that older Chinese Australians, with great language barriers, were not aware of the option to initiate ACP [39]. Complexed with a human nature to procrastinate EOL decision-making, it is unsurprising that while nearly 70% heard ACP discussions from our study, our subjects are illiterate in all ACP aspects. Even among those who are aware of ACP, lacking specific ACP knowledge has been shown to impact on readiness, receptiveness, and willingness to engage in ACP [13,22,43]. Our few contemplator subjects agreed that transitioning from awareness to action can be difficult, and this is why AD completions in this sample is much lower than the global norm.

Limited literacy presents a substantial barrier to ACP discussions [42], and for older Taiwanese’s program developers, the implication is to first increase public understanding (ACP literacy) and facilitate ACP discussions by providing related knowledge tailored to individual needs, such as explaining options of treatment and care in layman terms, appointing family surrogates, suggesting ACP clinics, giving opportunities for meaningful EOL discussion with providers, and documenting ADs and ACP discussions within the family. By offering proper ACP knowledge, a nurse-led education intervention has successfully increased Chinese-ethnic subjects’ AD completions and ACP discussions [45]. Once ACP was introduced to our subjects, we anticipate positive outcomes from ACP to improve compliance with patients’ EOL wishes, decrease subsequent hospitalization, lower death rates in hospitals and critical care units, and promote quality of deaths at home in the Taiwanese community.

#### 4.1.3. Influence of a Collectivistic Culture and Family Decision-Making on ACP Readiness

Across a number of countries where is ACP is promoted, ethnic minority people’s ACP readiness is generally low [46]: approximately 8–10% in Europe [4], 10–15% in Australia [47], and 10% or below in Asian countries. With a growing interest in the profound cultural influence on EOL decision-making [48], older, frail, and ethnic minority groups’ ACP readiness is low, due to low access to palliative care. The white and well educated who have a good familial support network and financial means engage better in ACP [7]. Our subjects’ low engagement can be explained by substantial family impact on treatment decision-making and a value conflict between individualistic and collectivistic decision-making.

Emerging literature shows that a shared decision-making model is desired among most racial and ethnic minorities, particularly in collectivistic cultures [49]. The individualistic model of ACP discussions, originated from western values, may not be culturally acceptable to non-white, Chinese-ethnic patients [22,23,31]. A 2012 immigrant study revealed that older Taiwanese American immigrants do not want to complete ADs without discussing with families [22]. Older Taiwanese expect ACP beyond the traditional patient–physician dyad to include important stakeholders in EOL discussions, such as family or community leaders [49]. Despite many Chinese-ethnic patients, particularly those of low-acculturation, value physician paternalism [22] and prefer a provider-based decision-making model [38], family members and community leaders remain Taiwanese people’s main source for medical decisions. In other words, the autonomous nature of ACP has fundamentally challenged Taiwanese’s preferred model of joint decisions with significant others in the family.

However, family decision-making may be controversial if patients’ wishes cannot be respected and followed. Multiple concerns exist in the shared decision-making model, such as decisional regret, non-disclosure of the diagnosis, value conflicts, and proxy anxiety. Highly influenced by Confucianism beliefs, self-efficacy for ACP is often limited among older individuals of Chinese cultural heritage [22,31,39] to defer medical decisions to physicians or family members. Our Taiwanese subjects also demonstrate little situational confidence when decisional conflicts rise among family members. This strong family influence combining with an uncertain about future illness often lead to ACP procrastination. Therefore, a common solution would be casually and verbally disclosing their EOL wishes to families rather than completing any formal ADs, similar to their overseas counterparts [22].

The reluctance of older Taiwanese’ family members to discuss EOL topics, regarded as a common ACP barrier among the older and frail group [50], is also apparent in our sample. Our subjects have passively expected that someone else will decide EOL treatment and care on their behalf. This low locus of control regarding EOL decisions [22,31], a salient Chinese cultural belief, also prevents older Taiwanese engaging in ACP. When disease progression and prognosis have been habitually informed only to family members, Taiwanese patients lose the opportunity to prepare for and engage in ACP [13].

Incorporating a familism value and cultural preferences into intervention programs will increase ACP discussions among diverse cultural groups, particularly in older Taiwanese. While the notion of familism is ingrained in Taiwan, family members remain important advocates to provide social and decisional support, facilitate communication with health care providers, and help overcome language barriers [22,51]. ACP programs should be designed within the context of family and community to include significant others as key decision-makers in ACP discussions. In particular, the role of the eldest son serving as the family head in medical decision-making should be recognized in modern Taiwanese society [13].

### 4.2. Recommendations of ACP Strategies for Older Taiwanese

#### 4.2.1. A Practical Nurse-Led Educational Intervention in the Community

What needs to be emphasized from our strategy findings are the implied challenges for program developers to design a practical and accessible ACP intervention in the community; in older subjects’ words, “something useful and worthy of my time.” Strategies older Taiwanese prefer are comparable to that of overseas Chinese Americans. A well-planned and structured educational program is considered the best facilitator of ACP behavioral change [22], and it will be more acceptable if ACP related knowledge can be effectively and efficiently delivered with up-to-date educational materials and resources.

An essential component of the program is to provide realistic guidance to maintain ongoing EOL communication within the Taiwanese family. With limited self-efficacy for ACP, a possible cultural taboo of death avoidance [31], and low ACP literacy, initiating ACP discussions is not easy in the Taiwanese culture. This leads to an integral implication to prepare community health care providers to “ice-break” and introduce EOL topics in daily conversations. Thoughtful, strategic ACP discussions will prepare older patients and families face advanced illness trajectory and cope with medical uncertainties in the future. Nurse-led ACP education in a community setting has been successful to increased AD knowledge and ACP engagement among Chinese Americans [38], and nurses in the community settings are often considered best candidates to deliver high-quality ACP discussions [52]. In particular, we believe those nurses who possess self-awareness, cultural knowledge, and effective communication skills should be equipped in the ACP front-line.

Since only very few health care professionals are formally trained to have ACP communication skills [52], a vast group of Taiwanese geriatric nurses working in the community clinics may serve as potential ACP advocators and educators. These easy-to-reach neighborhood professionals may build rapport with their older clients. While well-guided group discussions with facilitated personal sharing is older Taiwanese’s desired format to learn ACP, a small group rehearsal conducted by local nurses would encourage and prepare shy and unconfident older Taiwanese women to speaking up in the family. This leads to continuous education and training for nurses regarding ACP, and more studies are needed to examine the community nurse’s role in ACP [53].

#### 4.2.2. A Staged Planning to Utilize Cognitive and Emotional Strategies and Include Families

We believe “precontemplator-believers” is an appropriate readiness stage to increase ACP literacy [46], and community health care providers can be trained to initiate ACP with a staged approach. Based on our cultural knowledge, un-institutionalized community residents will openly discuss what they understand about and expect from life-sustaining treatment. While health literacy and socio-demographics are stronger predictors, providing easy-to-understand ACP materials will increase greatest ACP readiness at this point, regardless of their prior experience or ACP knowledge [37]. However, questions of “preferred place of death” or “artificial nutrition and hydration to be used at the EOL” can be saved till later from our cultural understanding. Our recommendation is consistent to program strategies suggested in a nationally acclaimed program “Respecting Choices” conducted among a majority of older Caucasians in a Midwest American community [54,55].

For those older patients with symptomatic chronic, progressive illness, we believe community health care providers may utilize clinical scenarios to facilitate clients developing a more detailed advanced care plan, including a timely goal to complete ADs, identify health care surrogates, even appoint durable power of attorneys for health care (DOPAs). In a collectivistic culture, this is a good time to involve clients’ significant others in ACP discussions, and scripts and decision algorithms may be used to remind the whole family of the EOL goals [54,55]. We do not recommend inexperienced community health care providers conducting ACP discussions for older Taiwanese patients who will most likely die within a year, since clear and transferable treatment orders should be made by trained professionals in specific ACP clinics in Taiwan. Applying “staged planning” to implement ACP is critically important for older Taiwanese who embrace Chinese culture and highly value familism at the precontemplation stage with low ACP readiness.

Regarding the commonly mentioned ACP barrier, human being’s procrastinating nature [56,57,58], our older subjects do not perceive imminent needs and view ACP less a life priority. Advanced plans do not reflect the actual medical, emotional, or social context in future EOL circumstances [59,60,61]. The implication is the usefulness of incorporating traditional cognitive and emotional strategies [36,62] in the programs to break the common myth that ACP is only practical among the actively dying. Older Chinese immigrants [22] found reflecting on real case studies, vivid clinical scenarios very helpful—relatively healthy ACP procrastinators were encouraged to considering the negative outcomes of postponement. We suggest a program strategy for older Taiwanese to discussing future adverse sequels in small groups in order to create a sense of urgency among their older peers. Probing questions could be, “Can you share with the group, if no ongoing ACP is in place or no EOL decisions are made, what is going to happen to you? Do you know anyone who has planned his or her EOL in advance? How is it working for him or her?”

#### 4.2.3. Cultural Specific Strategies for Chinese-Ethnic Older Taiwanese

We learned from our subjects that public media play an important role in shaping older Taiwanese’s death attitudes and impacting their awareness of EOL decision-making. Subjects reported EOL conversations usually occur in the family when watching life-support circumstances on TV or reading news about numerous deaths after natural disasters. Further market research will be valuable to understand what types of media older Taiwanese may be psychologically triggered to initiate ACP. Easy-to-access multi-media educational materials should be developed for older Taiwanese [63].

In our experience, older Chinese and Taiwanese demand respect and generally do not want to be coerced [13,18,22]. When introducing ACP, educators need to be patient without extra pressures to allow ACP behavioral changes to gradually occur [64]. On the other hand, to increase ACP uptake and intervention acceptability, poorly planned and less informative programs should be avoided. Transportation convenience, situational availability, and older subjects’ stamina and functioning abilities should be taken into serious consideration [22]. These practical suggestions of ACP programs corresponded to a recent ACP clinical guideline [23].

### 4.3. Study Strengthes and Limitations

#### 4.3.1. A Theory-Based Mixed-Method Pilot Study

Our study lays the theoretical groundwork for future ACP program development. In this pilot stage, efforts that begin with subjects’ self-identified facilitators and barriers are appropriate, as suggested by a clinical framework for improving the ACP process [65]. To facilitate positive stage transition, we first conceptualize interrelated (but separate) ACP behaviors [36], characterize behavioral engagement, and then explore socio-demographic determinants [66]. Since each stage of readiness leads to different patterns of behavioral changes in the ACP outcome [67,68,69], our work that conceptually differentiates and depicts ACP behaviors [70,71] is fundamentally important. While ACP research is scarce in Taiwan, our study also contributes valuable first-hand information regarding relatively healthy older community residents’ perceived factors and preferred program strategies.

Despite a modest sample size, findings from this study support our premise that the actual distribution of ACP engagement is highly skewed. Although evidence-based recommendations from the TTM research suggest dichotomizing the sample and focus on precontemplators, we recognize that the two groups do not fully represent the range of behavioral difference. Simplifying the ACP outcome reduces the outcome variance, leading to a possible lowering of correlation between ACP engagement and contributing factors. Larger community samples will statistically adjust this problem, and this is especially important when numbers in the advanced ACP stages (preparers and actioners) are expected to be low in Taiwan. In the future, interpretations of determinants should be taken into careful consideration if regressions analyses are to be performed.

More research is required to validate and justify the six-month cutoff point for older Taiwanese subjects to progress forward to each stage [29,30]. Our rather healthy subjects have difficulties projecting ACP behavioral changes within six months; in their own words, “it would be hard to imagine any (behavioral) difference since I am still quite okay.” When EOL situations are uncertain, facilitating ACP behavioral changes every six months is not realistic [23]; older Taiwanese will not actively seek for ACP information, enthusiastically complete AD documents, or constantly keep EOL communication if there exists no sudden change of health or no imminent death threats.

Last, our descriptive study employs a mixed-methods design to combine both quantitative and qualitative results to gain insight into the readiness phenomenon [13]; elements of themes that emerged in our subsequent interviews have augmented, enriched, and enhanced our survey findings. Interpretations from both types of findings offered a better generalizability [25,26] even when the pilot sample size is modest. The attempt to use a purposive community sample that seeks for annual geriatric health examinations is also advantageous. Older Taiwanese regularly participate in government-sponsored health care and visit local health facilities normally within a 20-mile radius. Our pilot study has demonstrated a cost-effective and time-efficient sampling technique to collaborate with a community health care facility. We recommend utilizing a convenient, up-to-date database of local geriatric residents that already exists. This recruitment strategy also permits program developers to establish problem areas in the community, identify and target potential subjects for future interventions, and provide insights for research directions for relatively healthy older Taiwanese [72].

#### 4.3.2. Sampling Biases and Future Research Directions

We recognize the inevitable bias of our non-probability, rather homogeneous sample; they are older, well-educated individuals physically transportable and accessible to the study site from a comparatively affluent metropolitan area in northern Taiwan. While not all eligible older Taiwanese had an equal chance to be included in this study, the selection bias limits the range and scope of our findings. In addition, significant effects explored from this modest-sized sample also attests to the robustness of our observed effects. For example, there may be correlational factors other than age and household income [6,7,43].

We strongly believe that minority groups’ voice should be heard [73], such as those physically challenged, uninformed patients who were bed-ridden, limited in mobility, and inaccessible to health care in the community. Future studies will benefit from larger samples by including Taiwanese of heterogeneous characteristics from a variety of settings to establish an external validity and the generalizable inference about ACP engagement. However, future challenges are present to explore ACP among critically ill [74], hospitalized [21], and institutionalized patients, residents in underdeveloped remote areas, foreign languages speakers, and seniors of relatively low socio-economic status. Ironically, these older community residents, inaccessible to health care, are those who need ACP the most.

In this pilot study, the relatively high level of openness in discussing death topics reflects only mainstream opinions or norms. While attained knowledge seems to be an important factor influencing ACP readiness [20], older Taiwanese in the minority group may have even lower sufficient awareness and ACP literacy, as informed from the mainstream culture. While older people in the minority group usually have little situational confidence to initiate EOL communication with family members [20,22,31], further investigations are required to explore the interaction between ACP awareness, candid communication, and behavioral engagement among minority Taiwanese patients of low socio-economic status who have limited health care resource.

Last, since causal conclusions cannot be drawn by our cross-sectional study, longitudinal designs are needed to examine the influence of cultural beliefs, knowledge, and adult children’s surrogate decision-making on ACP engagement over time, as well as how these factors change in response to the changing communication landscape around EOL decisions. Despite these limitations, this pilot study made an initiative to understand community-dwelling Taiwanese’s ACP engagement when they were still physically and cognitively intact to access and well utilize health resource covered by national insurance.

## 5. Conclusions

In conclusion, this mixed-methods study made an initiative to theoretically defined and assessed ACP engagement among older community-residing subjects in northern Taiwan. Socio-demographic correlates and influencing factors of program participation were explored, including facilitators, inhibitors, and useful intervention strategies preferred by this age group. In general, older Taiwanese of Chinese cultural heritage are at a fairly low stage of behavioral readiness; they perceived no imminent needs to proactively initiate ACP discussions and passively and infrequently communicated EOL decisions within the family. However, our results hold promise for older Taiwanese in the community to further engage in ACP. In contrast to the commonly known death taboos in Chinese culture, older subjects have openly discussed EOL decisions and provided rich information about ACP programs. Neighborhood out-patient geriatric clinics in Taiwan are potentials for conducting this type of community research, as well as for nurses to continue subsequent work to provide easy-to-access educational materials. Carefully designed interventions that apply “staged planning” principles, utilize clinical scenarios, and facilitate family-oriented ACP discussions will be essential to increase behavioral engagement and suit older Taiwanese’s multiple needs for ACP.

## Figures and Tables

**Table 1:** The stage of change for the Advance Care Planning algorithm.

Survey Questions	Precontemplation (Non-Believer)	Precontemplation (Believer)	Contemplation	Preparation	Action	Maintenance
Are you willing to start planning for your future life-sustaining treatment and possible care at the end-of-life (EOL)?	No, I don’t need to plan.	- I don’t know if I am willing to plan. - Yes, but I don’t know what time is better. - Yes, but later after 6 months.	Yes, soon within the next 6 months.	Yes, soon within the next 30 days.		
2. Do you want to know more about “Advance care planning (ACP)?”	No, I don’t need to know more.	- I don’t know if I need to know more. - Yes, but I don’t know what time is better. - Yes, but later after 6 months.	Yes, soon within the next 6 months.	Yes, soon within the next 30 days.		
3. In your opinion, do you think it is necessary now to plan for your future life-sustaining treatment?	No, not necessary.	I don’t know if it is necessary.	Yes, I think it is necessary.	Yes, I think it is necessary.		
4. Have you signed any advance directive?	No.	No.	No.	No.	Yes, less than 6 months.	Yes, longer than 6 months.
5. If you have signed an advance directive, have you told people that you have done so?					Yes, or no.	Yes.
6. If you have signed an advance directive, do you still talk to people about your decisions in your advance directive?					Yes, or no.	Yes.
7. If you have not signed an advance directive, are you able to sign on today?		- Yes. - I don’t know. - No.	- No. - I don’t know.	Yes.		

**Table 2:** Sample characteristics (N = 52).

Demographic Characteristics		N	%	Interviewed n ^1^
Age		52		
73.53 ± 6.98	65–74	29	55.8	4
	75–84	19	36.5	7
	≥85 years old	4	7.6	2
Gender		51		
	Male	22	43.1	6
	Female	29	56.9	7
Marital status		52		
	Single	2	3.8	1
	Married/partnered	34	65.4	7
	Widowed	15	28.8	5
	Divorced/separated	1	1.9	0
Parents alive		52		
	None of them alive	47	90.4	12
	One still alive	3	5.8	1
	Both alive	2	3.8	0
Education		52		
	Doctoral degree	5	9.6	1
	Master’s degree	8	15.3	0
	Bachelor’s degree	5	9.6	3
	College/Tech	10	19.2	4
	Senior High school	13	24.9	3
	Junior High school	11	21.2	2
Employment status		52		
	Disabled	0	0	0
	Retired	32	65.8	8
	Employed	6	11.5	1
	Housewife	8	15.3	3
	Unemployed	4	76	1
	Others	2	3.8	0
Household monthly income $17,645 ± $8998 ($1NT = $0.3 USD)		52		
	>$100,000	2	3.8	0
	$75,000 to $99,999	6	11.5	0
	$50,000 to $74,999	3	5.8	0
	$35,000 to $49,999	7	13.5	2
	$25,000 to $34,999	15	28.8	4
	$15,000 to $24,999	10	19.2	4
	$10,000 to $14,999	4	7.7	2
	$5000 to $9999	3	5.8	1
	<$5000	2	3.8	0
Religion		52		
	Atheist	4	7.7	0
	Buddhist	11	21.2	0
	Taoist/Taiwanese tradition	15	28.8	4
	Christianity (Catholics/Protestants)	10	19.2	4
	No preference	11	21.2	5
	Others	1	1.9	0

^1^ Interviewed subjects n = 13.

**Table 3:** Descriptives of the ordinal and dichotomous stages of readiness for advance care planning (ACP).

Stages of Readiness for ACP		N	%
Six ordinal stages (score determined by an algorithm)		52	
	Precontemplation (non-ACP believer)	2	3.8
	Precontemplation (ACP believer)	42	80.76
	Contemplation	5	9.61
	Preparation	2	3.84
	Action	1	1.92
	Maintenance	0	0
Two dichotomous stage (ordinal score recoded into 2 categories)		52	
	Precontemplators	44	84.6
	Contemplators, preparers, and actioners	8	15.38

**Table 4:** Correlation matrix of demographical variables and stages of readiness for ACP.

Demographical Characteristics	Six Ordinal Stages	Dichotomous Stages
Age	0.36 **	0.41 **
Gender	0.08	0.13
Life partner	−0.01	−0.09
Parents deceased	0.61 **	0.40 **
Education	0.05	0.09
Household income	0.02	0.23 *
Employment	−0.06	−0.07
Religion beliefs	0.04	0.04

* Correlation is significant at the 0.05 level (2-tailed); ** Correlation is significant at the 0.01 level (2-tailed).

**Table 5:** Intervention strategies older Taiwanese preferred in the ACP program.

Questions	Count (%)
Please tell us would you be interested in such a program? ^1^	
Yes	43 (82.6)
No	9 (17.3)
2. How would you prefer this program to be offered? ^1^	
Individually (one-on-one)	10 (19.2)
In a small group (5 people or fewer)	36 (69.3)
In a large group (6 people or more)	6 (11.5)
3. How long would you prefer each session to last? ^2^	
No more than 1 h	25 (48)
No more than 2 h	20 (38.4)
Four hours (with a break in between)	4 (7.6)
An entire day (more than 4 h with breaks in between)	2 (3.8)
Other; none of the above	1 (1.9)
4. How often would you be willing to come? ^3^	
Once a month	7 (14)
Every week	16 (32)
Every other week	6 (12)
Just once	20 (40)
Every 3 months	0
Every 6 months	0
Don’t care	1 (2)
Missing value	2 (3.8)
5. Persons preferred to participate with in the program (185 multiple choice counts)? ^3^	
No preference	44 (84.6)
Family members only	36 (69.2)
As long as I know who they are	2 (3.8)
Strangers only	28 (53.8)
People I know of the same gender	30 (57.6)
People I know and with similar ages	10 (19.2)
Nobody; self-learning	31 (59.6)
Other	3 (2.8)
Missing	3 (2.8)
6. Learning activities in the program (219 multiple choice counts)? ^1^	
Audiotapes/CD	49 (94.2)
Group discussion	40 (75.4)
Lecture	52 (100)
Work on the computer	21 (40.3)
Read written materials	48 (92.3)
Videotapes/DVD	4 (7.7)
Don’t care	5 (9.6)
7. Learning sites preferred in the program? (219 multiple choice counts)? ^3^	
In my church/temple	34 (66.6)
In the community, such as public a recreation center or a library	41 (80.4)
In a healthcare facility, such as hospitals or nursing homes	49 (96)
In a nearby school	22 (42.3)
Other/None of the above	6 (11.7)

^1,2^ n = 52, ^3^ n = 49.
